# Detection of hantavirus in bats from remaining rain forest in São Paulo, Brazil

**DOI:** 10.1186/1756-0500-5-690

**Published:** 2012-12-21

**Authors:** Jansen de Araujo, Luciano Matsumiya Thomazelli, Dyana Alves Henriques, Daniele Lautenschalager, Tatiana Ometto, Lilia Mara Dutra, Caroline Cotrin Aires, Sandra Favorito, Edison Luiz Durigon

**Affiliations:** 1 BSL3+ Laboratory, Instituto de Ciencias Biomedicas, Universidade de Sao Paulo, Sao Paulo, SP CEP: 05508-900, Brazil; 2 Museu de Zoologia, Universidade de Sao Paulo, Sao Paulo, Brazil; 3 Universidade Bandeirante de Sao Paulo - UNIBAN, Sao Paulo, Brazil

**Keywords:** Hantavirus, Wild rodents, Bats, Remaining rain forest, Brazil

## Abstract

**Background:**

The significant biodiversity found in Brazil is a potential for the emergence of new zoonoses. Study in some places of the world suggest of the presence to hantavirus in tissues of bats. Researches of hantavirus in wildlife, out rodents, are very scarce in Brazil. Therefore we decided to investigate in tissues of different species of wild animals captured in the same region where rodents were detected positive for this virus. The present work analyzed ninety-one animals (64 rodents, 19 opossums, and 8 bats) from a region of the Atlantic forest in Biritiba Mirin City, São Paulo State, Brazil. Lungs and kidneys were used for RNA extraction.

**Findings:**

The samples were screened for evidence of hantavirus infection by SYBR-Green-based real-time RT-PCR. Sixteen samples positive were encountered among the wild rodents, bats, and opossums. The detection of hantavirus in the lungs and kidneys of three marsupial species (*Micoureus paraguayanus*, *Monodelphis ihering*, and *Didelphis aurita*) as well in two species of bats (*Diphylla ecaudata* and *Anoura caudifer*) is of significance because these new hosts could represent an important virus reservoirs.

**Conclusions:**

The analysis of nucleotide sequences of the partial S segment revealed that these genes were more related to the Araraquara virus strains. This work reinforces the importance of studying hantavirus in different animal species and performing a continued surveillance before this virus spreads in new hosts and generated serious problems in public health.

## Findings

### Background

The principal natural known reservoirs of the hantavirus are wild rodents. Studies illustrate that rodents infected with hantaviruses are stable reservoirs, shedding the virus in urine, saliva and faeces, but do not show overt signs of disease [1]. The significant biodiversity found in Brazil is a potential for the emergence of new zoonoses as well as unprecedented invasion of human populations in previously uninhabited regions. Hantaviruses are a serious public health problem in developing country as Brazil. From 1993 through May 2012, a total of 1.573 cases of Hantavirus pulmonary syndrome (HPS) were reported in Brazil (case fatality rate 39%). Due to previous reports on the presence of hantavirus in tissues of bats [2,3] and the lack of hantavirus surveys in other wildlife out rodents, we decided to investigate the presence of this virus in tissues of different wildlife species captured in the same region where rodents have been detected positive for hantavirus.

In 2001, the region around Biritiba Mirin (a city in the state of São Paulo, Brazil) experienced severe environmental degradation by the clearing of approximately 11.4 km sq of rain forest. This area has been flooded for dam construction and the wildlife is forced onto smaller tracts of land concentrating the potential disease reservoirs. Additionally, other forest areas are being illegally cleared and used for agricultural purposes increasing contact between wild animals and humans and likewise the concern of health workers.

## Methods

The present work analyzed ninety-one animals (64 rodents, 19 opossums, and 8 bats) from a region of the Atlantic rain forest in Biritiba Mirin City (23°34’28´´ S -46°02’18´´W), Sao Paulo State, Brazil. Bats were captured using mist nets strategically placed between 30 cm and 2 meters high arranged in parallel inside the forest during the night. The rodents and opossums were captured using Sherman and Tomahawk traps following the methodology previously described [4]. The procedures involving measurements (total length, weight and evaluations of biometrics in general for species identification), collecting blood samples, and tissue removal were all performed inside a Bio Hazard Glove-Box containment cabinet (Micro Iso MK II, Envair Limited) with HEPA exhaust filters for biosafety procedures.

Lung, kidney, spleen, liver, and heart tissues as well as urine samples were taken from each animal, following protocols approved by the Ethics Committee on Animal Experimentation of the Institute of Biomedical Sciences, University of São Paulo and with the permission of Brazilian Institute of Environment and Renewable Natural Resources (IBAMA, process number 16575-1). All other laboratory manipulations were performed inside the BSL 3 following standard operational procedures [5]. For hantavirus detection was carried out by RT-PCR, real-time PCR and sequencing using conventional protocols to study hantavirus [3,4,6].

**Figure 1:**
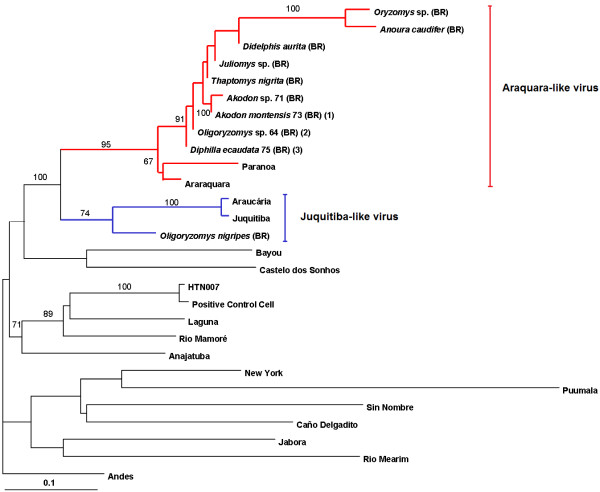
Dendrogram of the sequence comprising the S segment from hantavirus strains determined using the PAUP* 4.0b program on a Macintosh computer. A Neighbor-joining algorithm methods and the HKY-parameter model was used to generate a tree on the basis of nucleotide sequence differences in the 264-nt. Bootstrap values >50%, obtained from 1.000 pseudoreplicates of the analysis are shown for the branch points. The strain sequences under study in this paper are (BR) and are available on GenBank: *Oligoryzomys nigripes* [FJ402967], *Micoureus paraguayanus *66 [FJ402968], *Monodelphis ihering *72 [FJ402969], *Didelphis aurita *[FJ402970], *O. nigripes *36 [FJ402971], *Diphylla ecaudata *75 [FJ402972], *Diphylla ecaudata *76 [FJ402973], *O. nigripes *62 [FJ402974], *Akodon montensis *63 [FJ402975], *Oligoryzomys* sp. 64 [FJ402976], *Thaptomys nigrita* [FJ402977], *Juliomys *sp. [FJ402978], *Oryzomys *sp. [FJ402979], *Anoura caudifer *[FJ402980], *Akodon *sp. 71 [FJ402981], *Akodon montensis *73 [FJ402982]. Numbers inside parenthesis indicates the number of identical sequences. The following published S-segment sequences were included in the analysis (GenBank accession numbers): Puumala [AB297665], Bayou [L36929], Sin Nombre [NC005216], New York [U47135], Caño Delgadito [DQ285566], Anajatuba [DQ451829], Rio Mearim [DQ451828], Laguna Negra virus [AF005727], Rio Mamoré [U52136], HTN-007 [AF133254], Castelo dos Sonhos [AF307324], Andes [AF324902], Jabora virus [EF492471], Juquitiba [EF492472], Araucaria [AY740633], Araraquara [EF571895], Paranoa [EF576661].

RNA was extracted from the lungs and kidneys of all captured animals using RNA extraction kit (MagMax TM-96 RNA Isolation Kit, Ambion®, Inc., Austin, TX, EUA), then amplified and detected by SYBR Green-based real-time RT-PCR (Applied Biosystems, Foster City, CA, USA) using specific primers previously described to amplify 141 bp fragment of the S segment [4]. Samples of animals that had positive results by real-time PCR were amplified by traditional PCR too, using primers specific to S segment previously described [6], generating a larger amplicon of 264 bp. These fragments were sequenced using BigDye Terminator kit (Applied Biosystems, Foster City, CA, USA). Nucleotide sequence data were analyzed using Neighbor-Joining algorithm in order to evaluate the similarity between sequences obtained from the present study and hantavirus sequences deposited in GenBank. HKY-parameter model was defined by the program model test with the software package PAUP 4.0b10 [7]. Results of the bootstrap obtained from 1000 pseudoreplicates are shown for the branch points (Figure 1) [8]. The nucleotide percentage similarities were calculated using the *MegAlign* program of DNASTAR package (DNASTAR, Inc.).

### Results

Of the ninety-one animals examined, sixteen were positive (17.6%), including: ten rodents belonging to five genera (*Oligoryzomys nigripes*, *Oryzomys sp., Akodon sp., Tapthomys nigrita, Juliomys sp*.; three species of opossum (*Monodelphis ihering, Micoureus paraguayanus, Didelphis aurita*); two hematophagous bats (*Diphilla ecaudata*) and one nectivorous bat (*Anoura caudifer*) (Table 1).

**Table 1:** Detection of hantavirus by real-time PCR in samples from wild animals

**Order**	**Species**	**N° of positive results/N° tested**	**(%)**
Rodentia	*Oligoryzomys nigripes*	4/23	(17.4)
Rodentia	*Oryzomys sp.*	1/7	(14.3)
Rodentia	*Akodon sp.*	1/7	(14.3)
Rodentia	*Akodon montensis*	2/19	(10.5)
Rodentia	*Tapthomys nigrita*	1/2	(50)
Rodentia	*Juliomys sp.*	1/2	(50)
Rodentia	*Nectomys squamipes*	0/3	(0)
Rodentia	*Delomys sp.*	0/1	(0)
Didelphimorfia	*Monodelphis rubida*	0/1	(0)
Didelphimorfia	*Monodelphis ihering*	1/6	(16.6)
Didelphimorfia	*Marmosops incanus*	0/2	(0)
Didelphimorfia	*Micoureus paraguaianus*	1/3	(33.3)
Didelphimorfia	*Didelphis aurita*	1/5	(20)
Didelphimorfia	*Marmosops paulensis*	0/2	(0)
Chiroptera	*Diphylla ecaudata*	2/4	(50)
Chiroptera	*Artibeus lituratus*	0/2	(0)
Chiroptera	*Anoura caudifer*	1/2	(50)
Total		16/91	(17.6)

Sequences obtained in the present study shared 93 to 95% nucleotide identity with the S segment of the Araraquara virus and Paranoa virus, forming a group nearest (Araraquara-like virus). Accession numbers (GenBank): *Micoureus paraguayanus* 66 [FJ402968], *Monodelphis ihering* 72 [FJ402969], *Didelphis aurita* [FJ402970], *Diphylla ecaudata* 75 [FJ402972], *Diphylla ecaudata* 76 [FJ402973] and *Anoura caudifer* [FJ402980]. Only one sample derived from a rodent of the species *Oligoryzomys nigripes* segregated into a different clade, with 89% similarity to the Juquitiba virus and Araucaria virus (Juquitiba-like virus group). The high similarity between the strains and the formation of a clade indicates that they are very close and share a common ancestor.

### Discussion

Cases of human hantavirus infections continue to be reported in São Paulo State, and they represent an especially serious problem in newly occupied and deforested areas. The constant aggression against the natural environment, sudden climatic changes could alter the population densities of rodents and increase the rate of viral dissemination.

In 2003, two months after we finished the sampling, local newspapers (Biritiba Mirim and region), reported that a farm worker died from hantavirus pulmonary syndrome (HPS) in the same region where the material was collected for the present study.

Previously work performed to validate this molecular surveillance of hantavirus in natural reservoirs revealed the presence of hantavirus Juquitiba-like in one *O. nigripes* specimen where as Araraquara- like virus were found in *O. nigripes*, *A. montensis* and others species [4]. This result was different of previously works that suggest that each group of hantavirus is generally associated with one specific species of wild rodent. Until now, only wild rodents such as *Necromys lasiurus*, *O. nigripes* and *Akodon sp*. were considered potential natural reservoirs in Brazil [9].

Hantaviruses are highly host-specific [9]; infection spillovers to other rodent species are rare except under unusual circumstances [10,11]. However, some authors show that host-switch may be more frequent than thought [3,12].

The presence of hantavirus in tissues of bat was reported in Korea in 1993, with viral antigens being isolated from the lungs and kidneys of the bats *Rhinolophus ferrumequinum* and *Eptesicus serotinus*[2,13]. Recently it was reported that hantavirus RNA was detected in ethanol-fixed liver tissue from two banana pipistrelles (*Neoromicia nanus*), captured in West Africa, in June 2011 [3].

The detection of hantavirus in the lungs and kidneys of three marsupial species (*Micoureus paraguayanus*, *Monodelphis ihering*, and *Didelphis aurita*) as well in two species of bats (*Diphylla ecaudata* and *Anoura caudifer*) is of significance because these new hosts could represent an important virus reservoirs.

Transmission of hantavirus to different species could occur if virus replication was linked to the feeding behavior of these animals, or if contact due to overlapping habitat use increased a period after significantly. This region has experienced severe environmental degradation, the amount of space and food available to these rodent populations has been greatly reduced. Alterations in the equilibrium of rodent populations and in the dynamics of their interactions with humans can determine the occurrence of outbreaks. This situation has been intensified by wide-spread and severe changes in local ecosystems during the last few decades [14]. To make matters worse, this is a region in which rodents species from different biomes coexist.

The presence of opossum and bats in the same area where wild infected rodents circulate could be related to the dissemination of this virus by contact with the feces and urine of these rodents. Moreover, after the revolutionary breakthrough of isolating Hantaan strain virus, at least 14 viruses have been confirmed, and more than 40 species of small mammals have been identified as reservoirs or sources of hantavirus infection in the world [15].

### Conclusions

Our finds demonstrate the circulation of hantavirus in bats and opossum in Brazil and suggest an interspecies transmission that could be explained by a possible spillover of hantavirus among the wild animal populations of Biritiba Mirim, followed by shedding. This work reinforces the importance of studying hantavirus in different animal species and performing a continued surveillance before this virus spreads in new hosts and generated serious problems in public health.

## Competing interests

The authors declare that they have no competing interest.

## Authors’ contributions

AJ and TLM carried out the molecular genetic studies, participated in the sequence alignment and drafted the manuscript. HDA, LDA, OT, DLM, ACC and FS participated in the sequence alignment, collect of samples, and laboratory work. DEL conceived of the study, and participated in its design and coordination and helped to draft the manuscript. All authors read and approved the final manuscript.
